# Expansion of peripheral helper T cells in the peripheral blood of patients with chronic graft-versus-host disease

**DOI:** 10.1007/s00277-025-06663-w

**Published:** 2025-11-03

**Authors:** Yuanyuan Du, Xuefeng He, Kangkang Lv, Youzhen Ge, Mimi Xu, Li Wan, Meng Zhou, Huizhu Kang, Chengyuan Gu, Ruju Wang, Yue Han, Yongxia Wu, Depei Wu, Yuejun Liu

**Affiliations:** 1 National Clinical Research Center for Hematologic Diseases, Jiangsu Institute of Hematology, The First Affiliated Hospital of Soochow University, Suzhou, China; 2 Institute of Blood and Marrow Transplantation, Collaborative Innovation Center of Hematology, Soochow University, Suzhou, China; 3 Department of Transfusion Medicine, Wuhan Hospital of Traditional Chinese and Western Medicine, Tongji Medical College, Huazhong University of Science and Technology, Wuhan, China; 4 Department of Microbiology & Immunology, Medical College of Wisconsin, Milwaukee, USA

**Keywords:** Peripheral helper t cells, B cells, Allogeneic hematopoietic stem cell transplantation, Chronic graft-versus-host disease

## Abstract

Chronic graft-versus-host disease (cGVHD), presented as an autoimmune-like syndrome, is an important late complication and has become the leading cause of non-recurrent death after allogeneic hematopoietic stem cell transplantation (allo-HSCT). A cohort of 52 cGVHD patients were analyzed for the presence of Tph cells, circulating follicular helper T (cTfh) cells and B cells in peripheral blood mononuclear cells (PBMCs). We found decreased frequency of cTfh cells, but increased frequency of Tph cells in post-transplant patients compared to healthy controls (HCs). Higher percentage and absolute number of CCR2^+^Tph cells were observed in cGVHD patients. The percentage and absolute number of Tph cells decreased significantly with anti-cGVHD therapy. The percentage of Naive B cells, Breg cells and pre-GC B cells was negatively associated with Tph cells. The absolute number of plasma cells was positively associated with the absolute number of Tph cells. The percentage and absolute number of post-GC B cells were both positively associated with Tph cells expression. Collectively, our results showed that Tph cells are involved in the development of cGVHD and correlate with the severity of the disease.

**Supplementary Information:**

The online version contains supplementary material available at 10.1007/s00277-025-06663-w.

## Introduction

As one of the major complications in the late stage of allogeneic hematopoietic stem cell transplantation (allo-HSCT), chronic graft-versus-host disease (cGVHD) reduces the quality of life of post-transplant patients. cGVHD is an autoimmune-like syndrome, occurring 100 days or more after transplantation [1]. It often involves a variety of organs, including the liver, skin, eyes, mouth, gastrointestinal tract and lungs. The interaction of CD4^+^ T cells and B cells is the basis to initiate antibody responses during the clearance of pathogens and the development of autoimmune diseases. Autoantibody production and tissue fibrosis caused by the activation of B cells by donor allogeneic CD4^+^ T cells have been critically involved in cGVHD pathogenesis [2].

B cells have various functions such as presenting antigens, secreting antibodies and cytokines. Kuzmina et al. showed that more than half of patients with cGVHD had detectable anti-self or allogeneic antibodies [3]. Jin et al. also observed that antibodies produced by donor B cells were required for thymic and lymphatic damage in cGVHD mice [4]. In addition, liver and lung tissues of mouse cGVHD models as well as in biopsies from cGVHD patients, immunoglobulin deposition was also observed [5]. The presence of circulating immune complexes further suggests that B cells are involved in the pathology of cGVHD.

Characterized as CD4^+^ T cells expressing CXCR5 and programmed death-1 (PD-1), follicular helper T (Tfh) cells play a key role in promoting B cell proliferation in follicles by providing costimulatory signals through inducible T cell costimulator (ICOS), CD40 ligand (CD40L) [6–9]. In addition, Tfh cells secret cytokine IL-21 that promote the proliferation and differentiation of B cells in follicles and further formation of germinal center (GC), thereby assisting B-cell differentiation into plasma cells and memory B cells and leading to the development of cGVHD. Moreover, previous study has shown that there was a group of cells that have the same function as Tfh cells and are known as circulating Tfh (cTfh) cells [7].

Recently, a new population of CD4^+^ T cells were identified with B cell helper function, called “Peripheral helper T” (Tph) cells, in inflammatory tissues of patients with rheumatoid arthritis (RA) [10]. Tph cells function as B-cell helper by secreting cytokines such as IL-21, CXCL13, ICOS and MAF. Instead of expressing CXCR5, which localizes Tfh cells to lymphoid follicles, Tph cells express high levels of chemokine receptors, such as CCR2, CX3CR1, and CCR5, which direct cell migration to sites of inflammation [11]. Moreover, CD38 is an activation marker of Tph cells [12]. In addition to RA, Tph cells have been implicated in the pathogenies of many other autoimmune diseases, such as systemic lupus erythematosus (SLE), IgG4-related disease, type 1 diabetes (T1D) and primary biliary cholangitis [13–16]. The identification of Tph cells phenotype greatly expands the range of B cell helper T cells that can be evaluated as biomarkers for autoantibody-related diseases. Consistently, a recent study demonstrated that Tph cells are augmented in patients with cGVHD and correlate with the severity of cGVHD [17]. However, the activated (CD38^+^) Tph cells, Tph cells subsets and dynamics of Tph cells before and after anti-cGVHD therapy have not been well described.

Hence, we aimed to determine the presence of Tph cells in peripheral blood mononuclear cells (PBMCs) from patients with different severity of cGVHD after allo-HSCT. In addition, we studied the presence of cTfh cells and the activation of B cells in these patients and therefore define the correlation of Tph cells function with the activity of these immune cells in cGVHD patients. We also monitored the dynamic changes of cTfh and Tph cells, as well as the activation status and subsets of Tph cells. These studies demonstrated that Tph cells played important roles in human cGVHD and could be a potential biomarker for cGVHD development and target for cGVHD treatment in the future.

## Patients, materials, and methods

### Patients and data

Fifty-two cGVHD patients who underwent allo-HSCT between August 2017 and February 2021 at the Center for Hematopoietic Stem Cell Transplantation at the First Affiliated Hospital of Soochow University were included in this study. At the same time, fourteen healthy controls (HCs) and fifteen patients without cGVHD during the same period served as controls. The characteristics of 67 post-transplant patients are shown in Table 1.. Informed consent was obtained from the recipient for the collection of human peripheral blood. This study was approved by the ethics committee of the First Affiliated Hospital of Soochow University, and the trial was conducted in accordance with the Declaration of Helsinki. Most patients received the Bu/Cy conditioning regimen [busulfan 0.8 mg/kg intravenously (i.v.) every 6 h for 12 doses followed by cyclophosphamide (CTX) 1.8 g/m2/day for 2 days], with others receiving the total body irradiation (TBI)/Cy regimen (850 cGy daily for 1 day followed by CTX). Patients with matched unrelated donor transplantations and haploidentical related donor transplantations were given ATG (Thymoglobulin; Sangstat, Fremont, CA) 2.5 mg/kg/day for 4 days, given from day − 5 until day − 2. GVHD prophylaxis includes cyclosporine A (CsA) and short-term methotrexate (MTX) or a combination of CsA, short-term MTX and mycophenolate mofetil (MMF). The dosage of CsA was 3 mg/kg/day, i.v. from day − 9. MMF was administered orally, 0.5 g every 12 h from day − 9 to engraftment. The dosage of MTX was 15 mg/m^2^ administered i.v. on day + 1 and 10 mg/m^2^ on days + 3, +6, and + 11.

**Table 1.** Characteristics of cGVHD Patients (*n*=52)

Characteristics	*n* (%)
Number of Patients	67
Age (median, range) (years)	38 (14–62)
Sex (female/male)	28/39
Diagnosis	
Acute lymphoblastic leukemia	20 (29.8)
Acute myeloid leukemia	30 (44.8)
Myelodysplastic syndrome	10 (14.9)
Mixed phenotype acute leukemia	1 (1.5)
Others	6 (9.0)
Conditioning regimen	
Bu/Cy	64 (95.5)
TBI/Cy	3 (4.5)
ATG	
Yes	16 (23.9)
No	51 (76.1)
Donor type	
MSD	16 (23.9)
MUD	6 (9.0)
Haploidentical	45 (67.1)
Cell source	
PBSCs	42 (62.7)
PBSCs + BM	25 (37.3)
aGVHD	
Yes	22 (32.8)
No	45 (67.2)
cGVHD	
Non	15 (22.4)
Mild/moderate	37 (55.2)
Severe	15 (22.4)

Bu/Cy: Busulfan/cyclophosphamide; TBI/Cy: Total body irradiation/cyclophosphamide; ATG: antithymocyte globulin; MSD: matched sibling donor; MUD: matched unrelated donor; PBSCs: Peripheral blood stem cells; BM: Bone marrow; aGVHD: acute Graft-versus-Host Disease; cGVHD: chronic Graft-versus-Host Disease

Prior to starting anti-cGVHD therapy, patients receive a thorough assessment to determine the severity and extent of their cGVHD, including a physical examination, laboratory assessment and consultation without tissue biopsy results. The assessment of cGVHD is based on the cGVHD severity score system developed by the NIH in 2014 [18–20], which can ultimately determine the patients of cGVHD as mild, moderate, and severe. First-line treatment for cGVHD is glucocorticoids with or without calcineurin inhibitors such as prednisone ± CsA/tacrolimus. If first-line regimens are not effective, second-line regimens such as sirolimus, ruxolitinib, ibrutinib, imatinib, rituximab, MMF, MTX, etc. are used.

## Isolation of PBMCs and flow cytometric analysis

Peripheral blood samples from patients with active cGVHD, without cGVHD and HCs in the same period were collected. The median time between sample collection and hematopoietic stem cell transfusion in post-transplant patients was 11 (3–74) months. Blood samples anti-coagulated with EDTA were processed within 6 h of collection. PBMCs were sorted using density gradient centrifugation (Ficoll-Hypaque, GE Healthcare). The following fluorescence-labeled anti-human monoclonal antibodies were used for flow cytometric analysis: PE anti-human CD4 (RPA-T4), PE/Cy7 anti-human PD-1 (NAT105), FITC anti-human CXCR5 (J252D4), PE/CF594 anti-human CXCR3 (G025H7), BV421 anti-human CCR6 (G034E3), APC anti-human CD38 (HIT2), BV510 anti-human CCR2 (K036C2), APC anti-human CD127 (A019D5), FITC anti-human CD25 (BC96), FITC anti-human IgD (W18340F), PE/Cy7 anti-human CD38 (HB-7), APC anti-human CD27 (M-T271) and BV421 anti-human CD19 (HIB19) (Biolegend, San Diego, CA, USA). Data were acquired using FACS NovoCyte (ACEA Biosciences, San Diego, CA, USA) and analyzed using Novoexpress programme (Agilent, version 1.5.0).

### Statistical analysis

Comparisons between multiple independent samples in this study were performed using one-way ANOVA or Kruskal-Wallis H test, and results are expressed as Mean ± SEM or quartiles. A paired t-test was applied to compare patients with cGVHD before and after treatment. Associations between two variables were analyzed using Spearman’s correlation. R represents the Spearman correlation coefficient, with positive value indicating a positive correlation and negative value indicating a negative correlation. The diagnostic value of biomarkers was evaluated using ROC curves. SPSS 23.0 and GraphPad Prism 8 (GraphPad Software, SanDiego, CA) software were used for analysis and graphing. The significance level was set at *P* < 0.05. For all statistics, if *P* < 0.05, they were considered statistically significant (*), less than 0.01 or 0.001 were shown as ** or ***, respectively.

## Results

### Patients developed cGVHD with different severity and treatment response following allo-HSCT

Whether or not the patient receives a consistently stable donor implant requires monitoring the degree of donor-recipient chimerism using dynamic PCR-STR. Among 52 patients with cGVHD, 37 had mild/moderate cGVHD and 15 had severe cGVHD. Among 37 patients with mild/moderate cGVHD, 30 cases involved one organ (21 cases of skin, 2 cases of eye, 6 cases of liver, 1 case of gut), 3 cases involved two organs (both skin and mouth), and 4 cases involved three organs (2 cases of skin, mouth and eye, 1 case of skin, liver and eye, 1 case of liver, gut and kidney). Among 15 patients with severe cGVHD, 5 cases involved one organ (3 cases of skin, 2 cases of lung), 3 cases involved two organs (2 cases of skin and lung, 1 case of skin and liver), and 7 cases involved three or more organs (1 case of skin, mouth and eye, 1 case of skin, liver, eye and lung, 1 case of skin, mouth, eye and lung, 1 case of skin, gut, lung and liver, 2 cases of skin, liver, lung, eye and mouth, 1 case of skin, gut, mouth, lung and kidney) (Table 2). Among the 52 cGVHD patients, 22 patients improved after first-line treatment with glucocorticoids and/or CsA or tacrolimus, 22 patients improved after second-line treatment, but the remaining 8 patients showed no improvement in cGVHD symptoms after first- and second-line treatment, and 3 patients eventually died of cGVHD progress.

**Table 2:** Characteristics of cGVHD patients (*n* = 52)

	no cGVHD	mild/moderate cGVHD	severe cGVHD
**Sex** (number, female/male)	15 (5/10)	37 (18/19)	15 (5/10)
**Age** (median, range) (years)	39 (17, 62)	38 (14, 59)	38 (17, 59)
**Months from transplantation to sample collection**	7 (4, 74)	9 (3, 25)	24 (10, 49)
**Conditioning regimen**			
Bu/Cy	15	35	14
TBI/Cy	0	2	1
**Donor type**			
MSD	3	5	8
MUD	2	4	0
Haploidentical	10	28	7
**Cell source**			
PBSCs	9	7	7
PBSCs and CBSCs	3	13	3
PBSCs and BMSCs	3	7	3
PBSCs, BMSCs and CBSCs	0	10	2
**aGVHD**			
Yes	4	15	3
No	11	22	12
**Organ affected by cGVHD**			
**One organ**			
Skin		21	3
Eye		2	0
Mouth		0	0
Lung		0	2
Liver		6	0
Gut		1	0
Kidney		0	0
**Two organs**			
Skin and mouth		3	0
Skin and lung		0	2
Skin and liver		0	1
**Three or more organs**			
Skin, mouth and eye		2	1
Skin, liver and eye		1	0
Liver, gut and kidney		1	0
Skin, liver, eye and lung		0	1
Skin, mouth, eye and lung		0	1
Skin, gut, lung and liver		0	1
Skin, liver, lung, eye and mouth		0	2
Skin, gut, mouth, lung and kidney		0	1

Bu/Cy: Busulfan/cyclophosphamide; TBI/Cy: Total body irradiation/cyclophosphamide; MSD: matched sibling donor; MUD: matched unrelated donor; PBSCs: Peripheral blood stem cells; CBSCs: Cord blood stem cells; BMSCs: Bone marrow stem cells; aGVHD: acute Graft-versus-Host Disease; cGVHD: chronic Graft-versus-Host Disease

### Differential presence of circulating Tph and Tfh populations in cGVHD patients

**Fig. 1:**
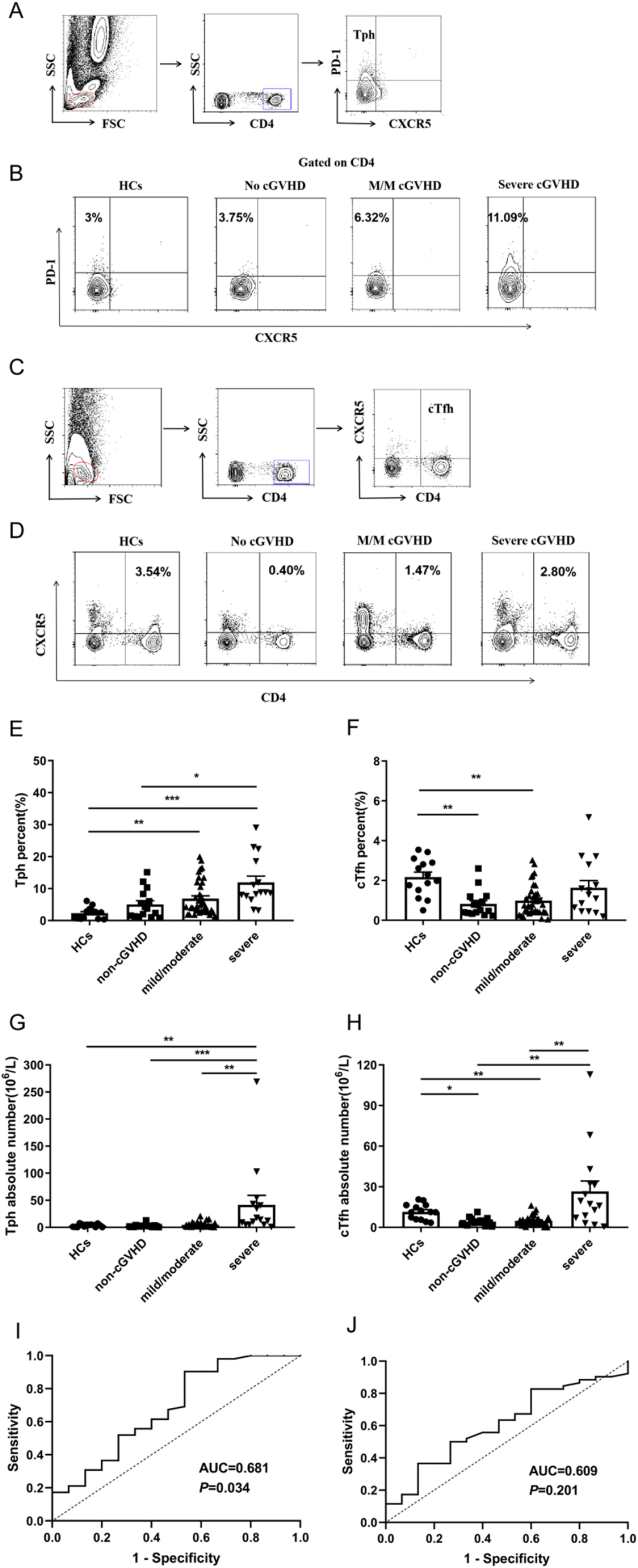
Expression of Tph cells and cTfh cells in HCs, non-cGVHD, mild/moderate and severe cGVHD patients. (**A**) Representative plots indicating gating strategy for identifying Tph cells (CD4^+^CXCR5^−^PD-1^+^). (**B**) Flow Cytometry (FCM) pattern of Tph (CD4^+^CXCR5^−^PD-1^+^) cells in HCs, non-cGVHD, mild/moderate and severe cGVHD patients. (**C**) Representative plots indicating gating strategy for identifying cTfh cells (CD4^+^CXCR5^+^). (**D**) FCM pattern of cTfh (CD4^+^CXCR5^+^) cells in HCs, non-cGVHD, mild/moderate and severe cGVHD patients. (**E**) The percentage of Tph cells was detected in the peripheral blood of HCs and patients with non-cGVHD, mild/moderate and severe cGVHD. (**F**) The percentage of cTfh cells was detected in the peripheral blood of HCs and patients with non-cGVHD, mild/moderate and severe cGVHD. (**G**) The absolute number of Tph cells was detected in the peripheral blood of HCs and patients with non-cGVHD, mild/moderate and severe cGVHD. (**H**) The absolute number of cTfh cells was detected in the peripheral blood of HCs and patients with non-cGVHD, mild/moderate and severe cGVHD. (**I**) The diagnostic value of Tph cells in non-cGVHD and cGVHD patients was evaluated using ROC curve. (**J**) The diagnostic value of cTfh cells in non-cGVHD and cGVHD patients was evaluated using ROC curve. Values are presented as quartiles. * *P* < 0.05; ** *P* < 0.01; *** *P* < 0.001

PBMCs from post-transplant patients and HCs were collected and stained for CD4, CXCR5 and PD-1 for analyzing Tph and cTfh populations. To study the correlation of Tph cells with human cGVHD, we analyzed the presence of Tph cells in the peripheral blood of post-transplant patients with the phenotype CD4^+^CXCR5^−^PD-1^+^ using flow cytometry (Fig. 1A and B). The percentage of Tph cells from allo-HSCT patients with mild/moderate cGVHD or severe cGVHD was significantly increased compared with HCs (4.38% versus 2.64%, *P* = 0.006; 8.81% versus 2.64%, *P*<0.001) (Fig. 1E). The severe cGVHD group had significantly higher absolute number of Tph cells than HCs (14.22*10^6^/L versus 3.98*10^6^/L, *P* = 0.002) (Fig. 1G). We next investigated whether the level of Tph cells in post-transplant patients correlated with disease severity, thus dividing the post-transplant patients into without cGVHD, mild/moderate cGVHD and severe cGVHD groups according to NIH consensus criteria [18–20]. The medium value of Tph cells percentage in patients without cGVHD, mild/moderate cGVHD and severe cGVHD are 3.75%, 4.38% and 8.81%, respectively (Fig. 1E). The percentage of Tph cells was significantly higher in patients with severe cGVHD than in those without cGVHD (*P* = 0.013) (Fig. 1E). The absolute number of Tph cells in patients without cGVHD, mild/moderate cGVHD and severe cGVHD was 2.03*10^6^/L, 3.04*10^6^/L and 14.22*10^6^/L, respectively (Fig. 1G). The absolute number of Tph cells was significantly higher in patients with severe cGVHD than in those without cGVHD and mild/moderate cGVHD (*P*<0.001; *P* = 0.002) (Fig. 1G).

In addition, we also analyzed cTfh cells characterized as CD4^+^CXCR5^+^ in HCs and post-transplant patients (Fig. 1C and D). The percentage of cTfh cells in patients without cGVHD, mild/moderate cGVHD and severe cGVHD showed a trend of increase according to severity of cGVHD (0.51%, 0.79% and 1.28%, respectively), but did not reach statistical significance (Fig. 1F). Interestingly, the percentage of cTfh cells was decreased in post-transplant patients compared to HCs. Among them, the percentage of cTfh cells in patients with non-cGVHD and mild/moderate cGVHD was significantly lower than that in HCs, and the differences were statistically significant (0.51% versus 2.14%, *P* = 0.001; 0.79% versus 2.14%, *P* = 0.002) (Fig. 1F). Meanwhile, the absolute number of cTfh cells in patients with non-cGVHD and mild/moderate cGVHD was significantly lower than that in HCs (3.60*10^6^/L versus 11.26*10^6^/L, *P* = 0.010; 3.87*10^6^/L versus 11.26*10^6^/L, *P* = 0.003) (Fig. 1H). The absolute number of cTfh cells in severe cGVHD patients was extremely increased than in patients without cGVHD or mild/moderate cGVHD (17.16*10^6^/L versus 3.60*10^6^/L, *P* = 0.004; 17.16*10^6^/L versus 3.87*10^6^/L, *P* = 0.001) (Fig. 1H).

When comparing patients with or without cGVHD, the cTfh cells cut-off value was 95.5% from the ROC curve, and the sensitivity and specificity were 50% and 73.3%, respectively (Fig. 1J). The AUC of Tph cells were 0.681 (95% CI 0.516–0.846, *P* = 0.034) (Fig. 1I). The Tph cells cut-off value was 2.43% from the ROC curve, and the sensitivity and specificity were 90.38% and 46.67%, respectively (Fig. 1I).

Taken together, our data indicated that Tph cells may be involved in pathogenicity of cGVHD, which is related to disease severity.

### Increased activated (CCR2^+^) Tph cells in cGVHD patients

**Fig. 2:**
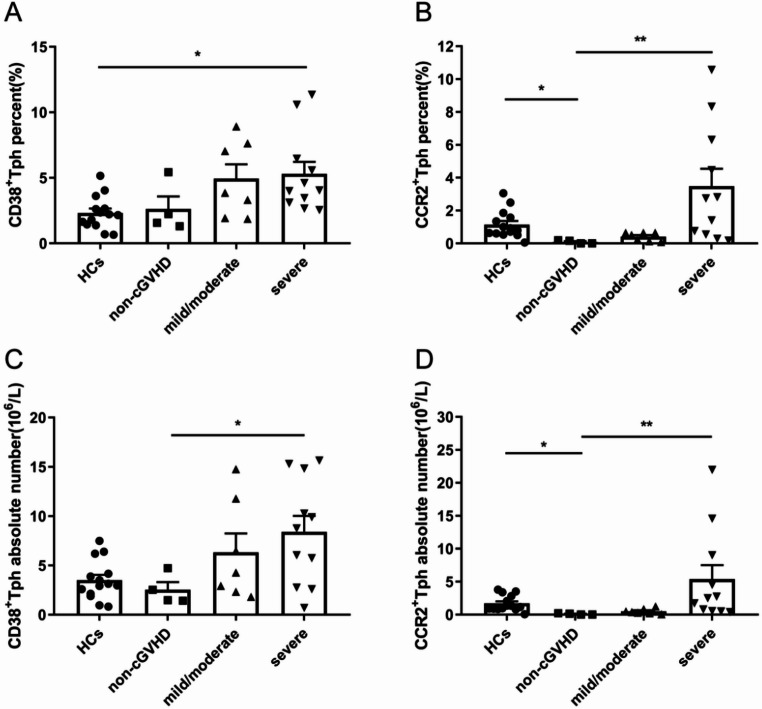
Expression of CD38^+^Tph cells and CCR2^+^Tph cells in HCs, non-cGVHD, mild/moderate and severe cGVHD patients. (**A**) The percentage of CD38^+^Tph cells was detected in the peripheral blood of HCs and patients with non-cGVHD, mild/moderate and severe cGVHD. (**B**) The percentage of CCR2^+^Tph cells was detected in the peripheral blood of HCs and patients with non-cGVHD, mild/moderate and severe cGVHD. (**C**) The absolute number of CD38^+^Tph cells was detected in the peripheral blood of HCs and patients with non-cGVHD, mild/moderate and severe cGVHD. (**D**) The absolute number of CCR2^+^Tph cells was detected in the peripheral blood of HCs and patients with non-cGVHD, mild/moderate and severe cGVHD. Values are presented as quartiles or Mean ± SEM. * *P* < 0.05; ** *P* < 0.01

CD38 and CCR2 are key markers for Tph cells activation and migration. In order to further explore Tph cells activation, we examined the expression of CD38 and CCR2 molecules on Tph cells from 22 allo-HSCT patients and 14 HCs. We found a higher percentage of CD38^+^Tph cells in patients with severe cGVHD than in HCs (4.05% versus 2.19%, *P* = 0.011) (Fig. 2A). The percentage of CD38^+^Tph cells in patients without cGVHD, mild/moderate cGVHD and severe cGVHD showed a trend of increase according to severity of cGVHD, but were not statistically different (1.90%, 3.86% and 4.05%, respectively) (Fig. 2A). The absolute number of CD38^+^Tph cells in patients without cGVHD, mild/moderate cGVHD and severe cGVHD also showed a trend of increase (2.53*10^6^/L ± 0.77*10^6^/L, 6.33*10^6^/L ± 1.91*10^6^/L and 8.41*10^6^/L ± 1.61*10^6^/L, respectively), and the difference between the severe and non-cGVHD group was statistically significant (*P* = 0.035) (Fig. 2C). In contrast, a higher percentage and absolute number of CCR2^+^Tph cells were found in HCs than in patients without cGVHD (0.86% versus 0.08%, *P* = 0.038; 1.25*10^6^/L versus 0.07*10^6^/L, *P* = 0.012) (Fig. 2B and D). The medium value of CCR2^+^Tph cells percentage in patients without cGVHD, mild/moderate cGVHD and severe cGVHD were 0.08%, 0.51% and 2.74%, respectively (Fig. 2B). The absolute number of CCR2^+^Tph cells in patients without cGVHD, mild/moderate cGVHD and severe cGVHD were 0.07*10^6^/L, 0.39*10^6^/L, 2.49*10^6^/L, respectively (Fig. 2D). Moreover, the percentage and absolute number of CCR2^+^Tph cells were significantly higher in patients with severe cGVHD than in those without cGVHD (*P* = 0.004; *P* = 0.005) (Fig. 2B and D). In addition, the percentage and absolute number of CCR2^+^Tph cells were slightly higher in patients with severe cGVHD than in those with mild/moderate cGVHD (*P* = 0.058; *P* = 0.078) (Fig. 2B and D). These data suggest that Tph cells are more activated as indicated by CCR2 expression in cGVHD patients, which correlates with disease severity. Unfortunately, the role of CD38^+^Tph cells in cGVHD requires further study with increased sample size.

### Expression of Tph cells subsets in peripheral blood of cGVHD patients

**Fig. 3:**
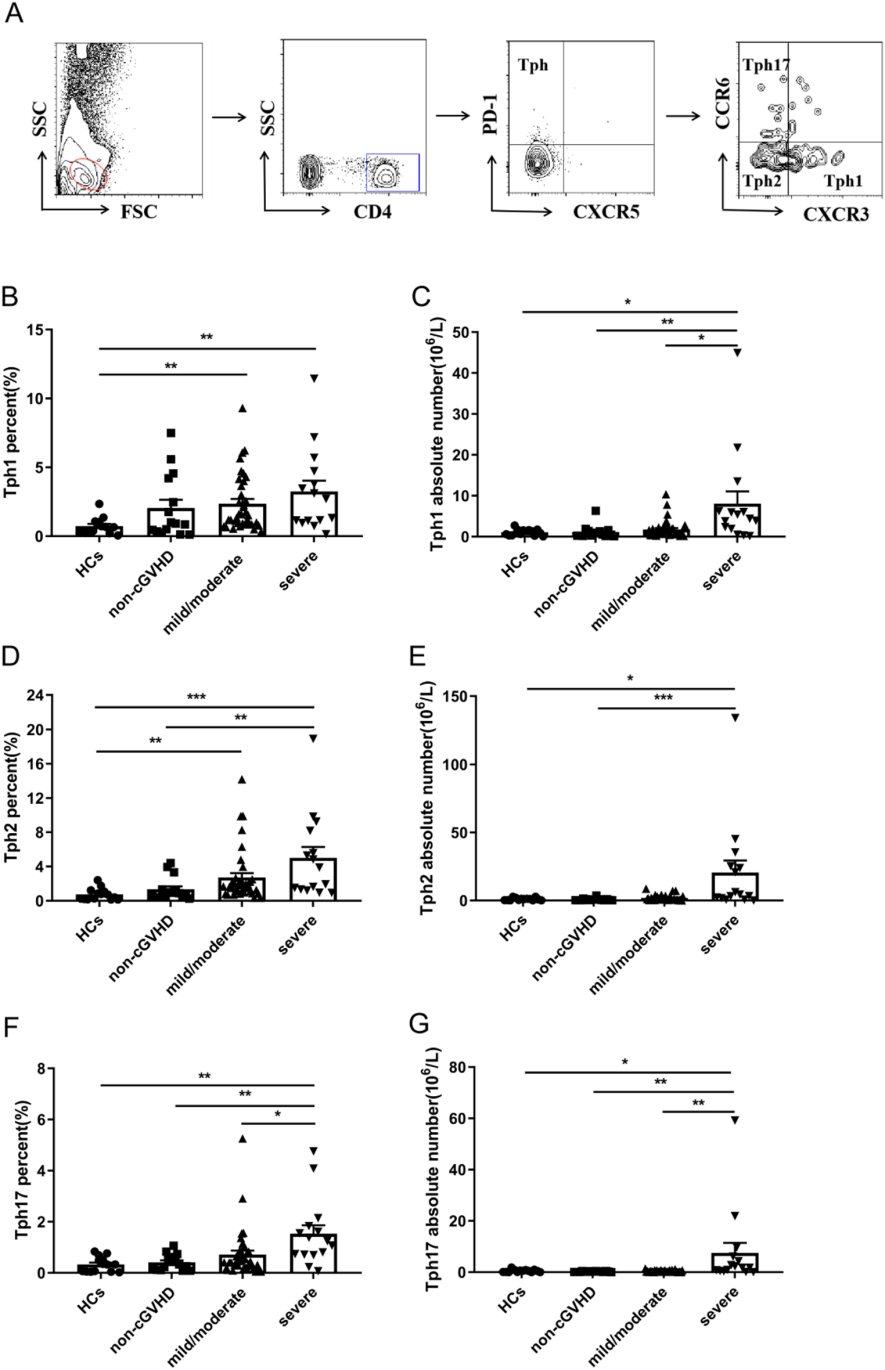
Tph cells subsets expression in the peripheral blood of HCs, non-cGVHD, mild/moderate and severe cGVHD patients. (**A**) Representative plots indicating gating strategy for identifying Tph cells subsets. (**B**) The percentage of Tph1 cells was detected in the peripheral blood of HCs and patients with non-cGVHD, mild/moderate and severe cGVHD. (**C**) The absolute number of Tph1 cells was detected in the peripheral blood of HCs and patients with non-cGVHD, mild/moderate and severe cGVHD. (**D**) The percentage of Tph2 cells was detected in the peripheral blood of HCs and patients with non-cGVHD, mild/moderate and severe cGVHD. (**E**) The absolute number of Tph2 cells was detected in the peripheral blood of HCs and patients with non-cGVHD, mild/moderate and severe cGVHD. (**F**) The percentage of Tph17 cells was detected in the peripheral blood of HCs and patients with non-cGVHD, mild/moderate and severe cGVHD. (**G**) The absolute number of Tph17 cells was detected in the peripheral blood of HCs and patients with non-cGVHD, mild/moderate and severe cGVHD. Values are presented as quartiles. * *P* < 0 0.05; ** *P* < 0.01; *** *P* < 0 0.001

Based on the expression of CXCR3 and chemokine C-C receptor (CCR) 6, Tph cells can be divided into 3 subsets: CXCR3^+^CCR6^−^ (Tph1) cells, CXCR3^−^CCR6^−^ (Tph2) cells, CXCR3^−^CCR6^+^ (Tph17) cells [21]. We then studied the different subsets of Tph cells in 67 post-transplant patients and 14 HCs. Representative plots indicating gating strategy for identifying Tph cells subsets are shown in Fig. 3A. Tph1 cells from patients with mild/moderate cGVHD or severe cGVHD were significantly increased compared with HCs (1.53% versus 0.67%, *P* = 0.006; 2.71% versus 0.67%, *P* = 0.004) (Fig. 3B). Additionally, the percentage of Tph1 cells were comparable in patients without cGVHD, mild/moderate cGVHD and severe cGVHD (Fig. 3B). In terms of the absolute number of Tph1 cells, the severe group was significantly higher than the HCs, non-cGVHD, and mild/moderate cGVHD group (4.40*10^6^/L versus 1.11*10^6^/L, *P* = 0.027; 4.40*10^6^/L versus 0.54*10^6^/L, *P* = 0.002; 4.40*10^6^/L versus 0.89*10^6^/L, *P* = 0.046, respectively) (Fig. 3C). The percentage and absolute number of Tph2 and Tph17 cells did not differ significantly between HCs and patients without cGVHD (Fig. 3D-G). Patients with severe cGVHD displayed significantly higher percentage of Tph2 cells than patients without cGVHD or HCs (3.88% versus 0.88%, *P* = 0.007; 3.88% versus 0.57%, *P*<0.001) (Fig. 3D). At the same time, the absolute number of Tph2 cells was higher in the severe cGVHD group than in the HCs and non-cGVHD group, and the differences were statistically significant (4.39*10^6^/L versus 1.01*10^6^/L, *P* = 0.010; 4.39*10^6^/L versus 0.43*10^6^/L, *P*<0.001) (Fig. 3E). The percentage of Tph2 cells were significantly higher in patients with mild/moderate cGVHD than in HCs (1.71% versus 0.57%, *P* = 0.002) (Fig. 3D). However, there was no statistical difference in absolute number between the two groups (Fig. 3E). The absolute number of Tph2 cells was slightly higher in the severe cGVHD patients than in the mild/moderate cGVHD patients (4.39*10^6^/L versus 1.10*10^6^/L, *P* = 0.061) (Fig. 3E). Interestingly, the percentage of Tph17 cells was also notably increased in patients with severe cGVHD than those in mild/moderate or without cGVHD or HCs groups (1.24% versus 0.45%, *P* = 0.025; 1.24% versus 0.33%, *P* = 0.009; 1.24% versus 0.33%, *P* = 0.001, respectively) (Fig. 3F). Meanwhile, the absolute number of Tph17 cells was also significantly increased in the severe cGVHD patients than in the HCs, non-cGVHD, and mild/moderate cGVHD groups (1.79*10^6^/L versus 0.52*10^6^/L, *P* = 0.047; 1.79*10^6^/L versus 0.24*10^6^/L, *P* = 0.001; 1.79*10^6^/L versus 0.25*10^6^/L, *P* = 0.004, respectively) (Fig. 3G). In summary, these data demonstrated the involvement of Tph2 and Tph17 cells in cGVHD pathogenesis, suggesting that the imbalance of Tph cells subsets may play important role during the onset of cGVHD.

### Effect of anti-cGVHD treatment on circulating Tph and Tfh cells

**Fig. 4:**
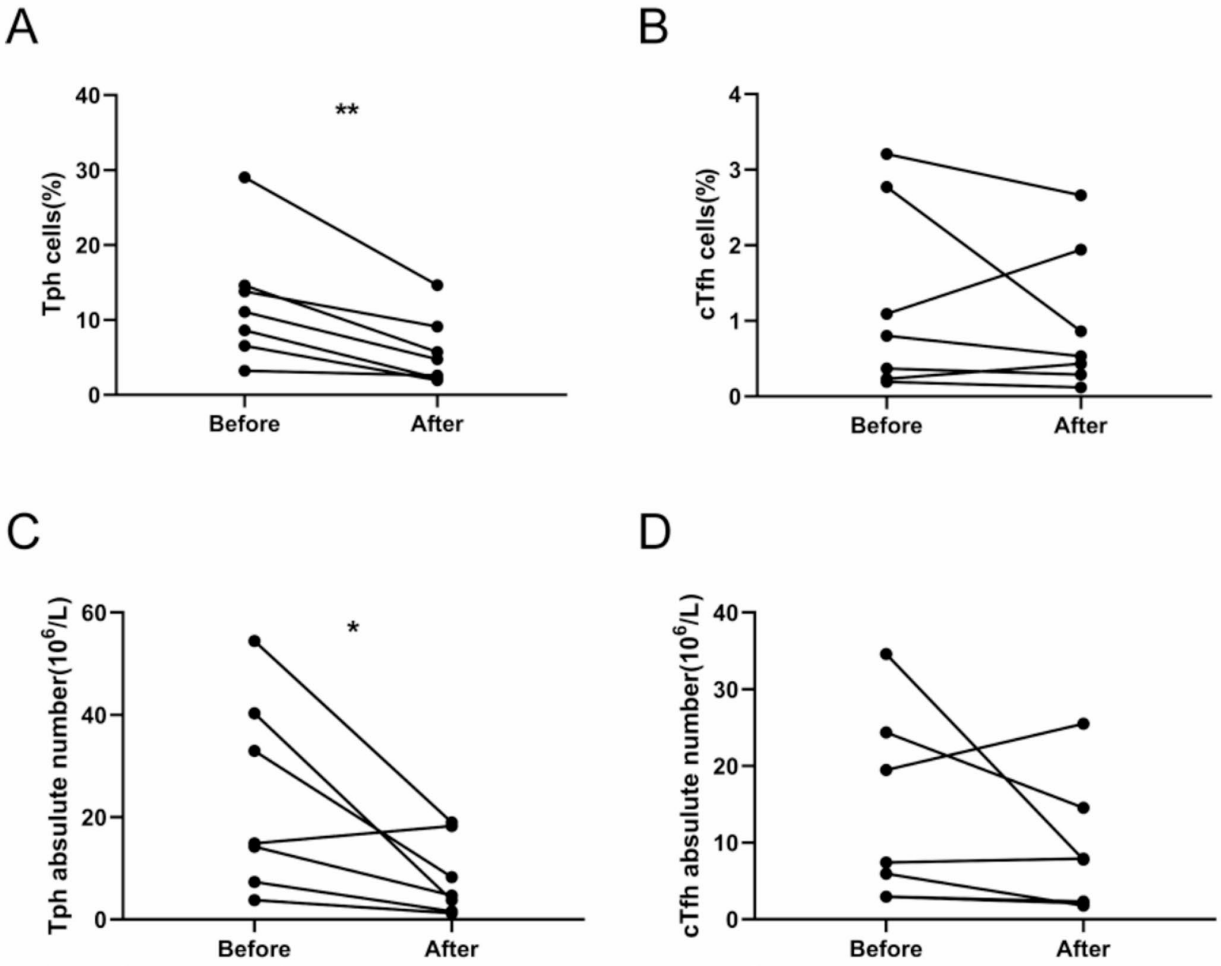
Dynamic changes of Tph cells and cTfh cells before and after anti-cGVHD treatment. (**A**) Graphs showing the percentage of Tph cells in cGVHD patients (*n* = 7) before and after anti-cGVHD treatment. (**B**) Graphs showing the percentage of cTfh cells in cGVHD patients (*n* = 7) before and after anti-cGVHD treatment. (**C**) Graphs showing the absolute number of Tph cells in cGVHD patients (*n* = 7) before and after anti-cGVHD treatment. (**D**) Graphs showing the absolute number of cTfh cells in cGVHD patients (*n* = 7) before and after anti-cGVHD treatment. * *P* < 0 0.05; ** *P* < 0.01

To investigate the dynamic changes of Tph and cTfh cells during the onset and control of cGVHD, seven cGVHD patients were re-evaluated after anti-cGVHD treatment. Of note, with the improvement of patients’ symptoms by anti-cGVHD therapy, marked decreases of the Tph cells percentage and absolute number were observed in all seven patients (*P* = 0.007; *P* = 0.041) (Fig. 4A and C). However, the percentage and absolute number of cTfh cells were comparable before and after anti-cGVHD treatment (*P* = 0.446; *P* = 0.254) (Fig. 4B and D).

Collectively, these results demonstrated that Tph cells were closely related to the development of cGVHD.

### Altered B cells subsets in the peripheral blood of patients who developed cGVHD after allo-HSCT

**Fig. 5:**
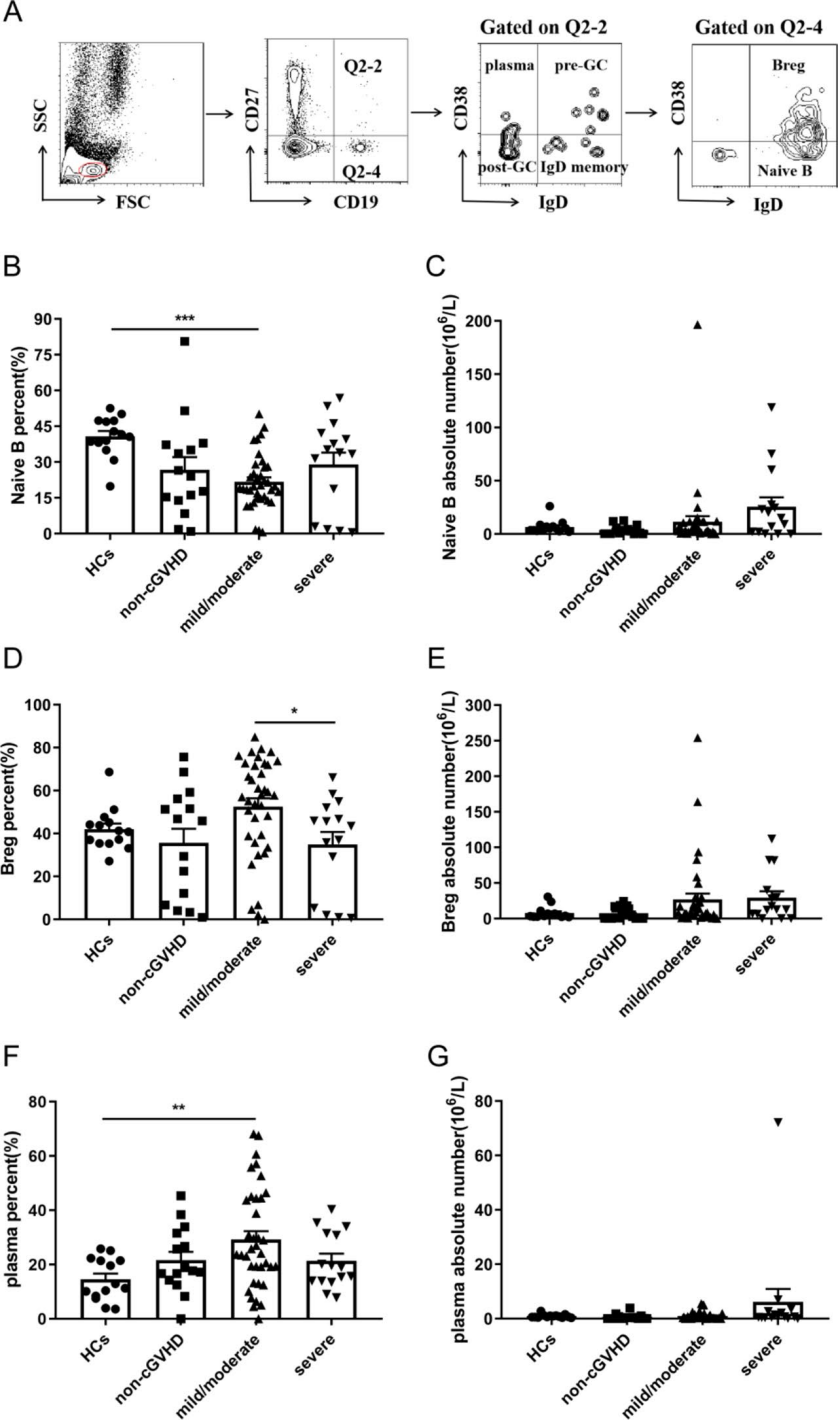

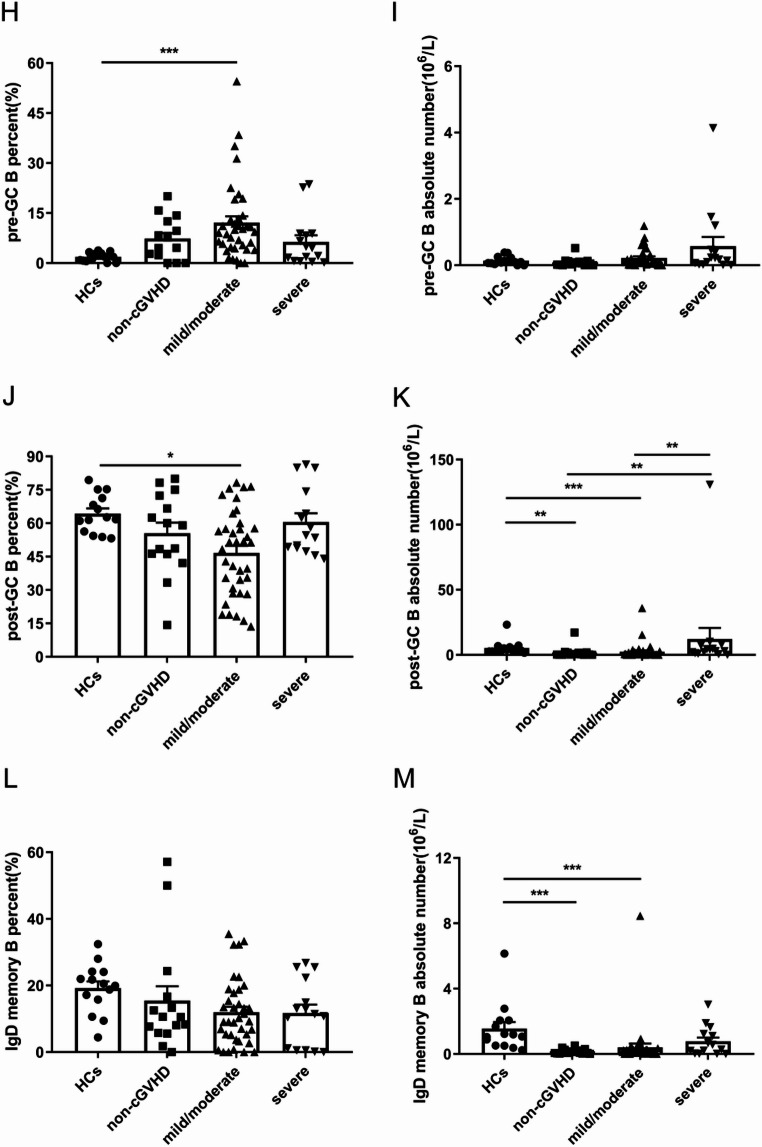
Expression of various subsets of B cells in HCs, non-cGVHD, mild/moderate and severe cGVHD patients. (**A**) Representative plots indicating gating strategy for identifying B cells subsets. (**B**) The percentage of Naive B cells was detected in the peripheral blood of HCs and patients with non-cGVHD, mild/moderate and severe cGVHD. (**C**) The absolute number of Naive B cells was detected in the peripheral blood of HCs and patients with non-cGVHD, mild/moderate and severe cGVHD. (**D**) The percentage of Breg cells was detected in the peripheral blood of HCs and patients with non-cGVHD, mild/moderate and severe cGVHD. (**E**) The absolute number of Breg cells was detected in the peripheral blood of HCs and patients with non-cGVHD, mild/moderate and severe cGVHD. (**F**) The percentage of plasma cells was detected in the peripheral blood of HCs and patients with non-cGVHD, mild/moderate and severe cGVHD. (**G**) The absolute number of plasma cells was detected in the peripheral blood of HCs and patients with non-cGVHD, mild/moderate and severe cGVHD. (**H**) The percentage of pre-GC B cells was detected in the peripheral blood of HCs and patients with non-cGVHD, mild/moderate and severe cGVHD. (**I**) The absolute number of pre-GC B cells was detected in the peripheral blood of HCs and patients with non-cGVHD, mild/moderate and severe cGVHD. (**J**) The percentage of post-GC B cells was detected in the peripheral blood of HCs and patients with non-GVHD, mild/moderate and severe cGVHD. (**K**) The absolute number of post-GC B cells was detected in the peripheral blood of HCs and patients with non-cGVHD, mild/moderate and severe cGVHD. (**L**) The percentage of IgD memory B cells was detected in the peripheral blood of HCs and patients with non-cGVHD, mild/moderate and severe cGVHD. (**M**) The absolute number of IgD memory B cells was detected in the peripheral blood of HCs and patients with non-cGVHD, mild/moderate and severe cGVHD. Values are presented as Mean ± SEM or quartiles. * *P* < 0.05; ** *P* < 0.01; *** *P* < 0.001

The development of cGVHD is closely associated with altered B cells subsets [4, 5, 22]. Based on the definition of B cells subsets in previous studies of various autoimmune diseases, B cells are classified as CD27^−^ B cells and CD27^+^ B cells indicating a subset of B cells that experienced antigen stimulation [23–25]. The Naive B, Breg, plasma, pre-GC B, post-GC B, IgD memory B cells subsets were further defined based on the expression of the surface antigens IgD and CD38. B cells subsets phenotypes are shown in Supplementary Table S1. Representative plots indicating gating strategy for identifying B cells subsets are shown in Fig. 5A. Naive B cells, Breg cells, post-GC B cells and IgD memory B cells from patients without cGVHD were decreased compared with HCs, none of the differences were statistically significant (Fig. 5B, D, J and L). On the contrary, patients without cGVHD showed slightly increases in the percentage of plasma cells and pre-GC B cells compared with HCs (Fig. 5F and H). We had not detected much differences regarding the percentage of various subsets of B cells among different subgroups of patients after transplantation. In terms of absolute number, there was no statistically significant expression between the various groups of Naive B, Breg, plasma, and pre-GC cells (Fig. 5C, E, G and I). For the absolute number of post-GC B cells, the severe cGVHD group was significantly increased than the non-cGVHD as well as mild/moderate cGVHD group (2.94*10^6^/L versus 0.45*10^6^/L, *P* = 0.008; 2.94*10^6^/L versus 0.51*10^6^/L, *P* = 0.007) (Fig. 5K). In addition, the absolute number of post-GC B cells was significantly decreased in the group with non-cGVHD as well as mild/moderate cGVHD than in the group with HCs (0.45*10^6^/L versus 3.34*10^6^/L, *P* = 0.001; 0.51*10^6^/L versus 3.34*10^6^/L, *P*<0.001) (Fig. 5K). The absolute number of IgD memory B cells was significantly increased in the HCs group than in the non-cGVHD group as well as mild/moderate group (1.13*10^6^/L versus 0.07*10^6^/L, *P*<0.001; 1.13*10^6^/L versus 0.13*10^6^/L, *P*<0.001) (Fig. 5M). In conclusion, the percentage and absolute number of the various subsets of B cells could not bring us any insights.

### Correlation of Tph cells expression with B cells subsets in peripheral blood of cGVHD patients

**Fig. 6:**
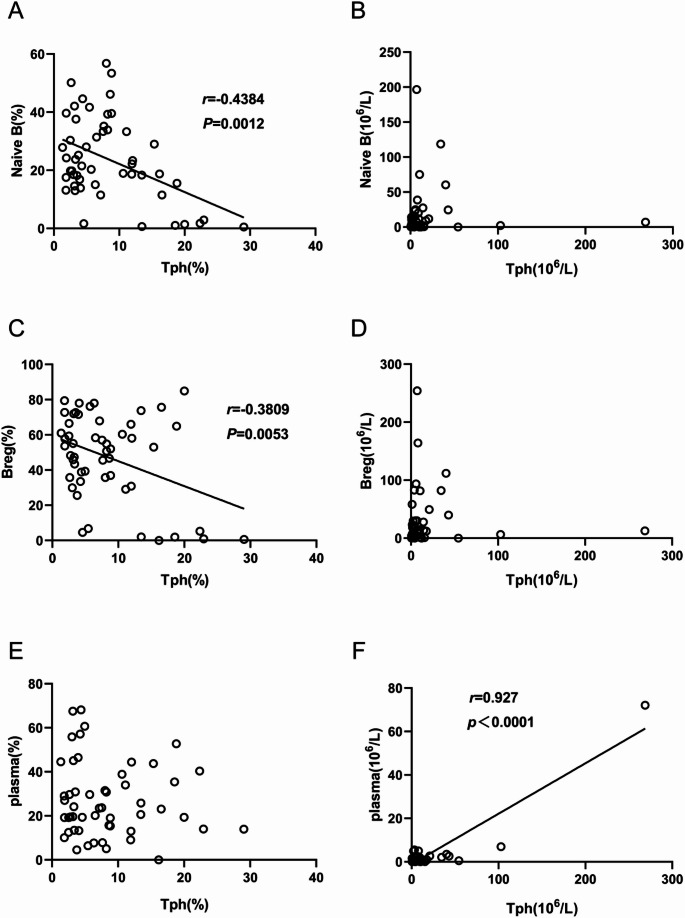

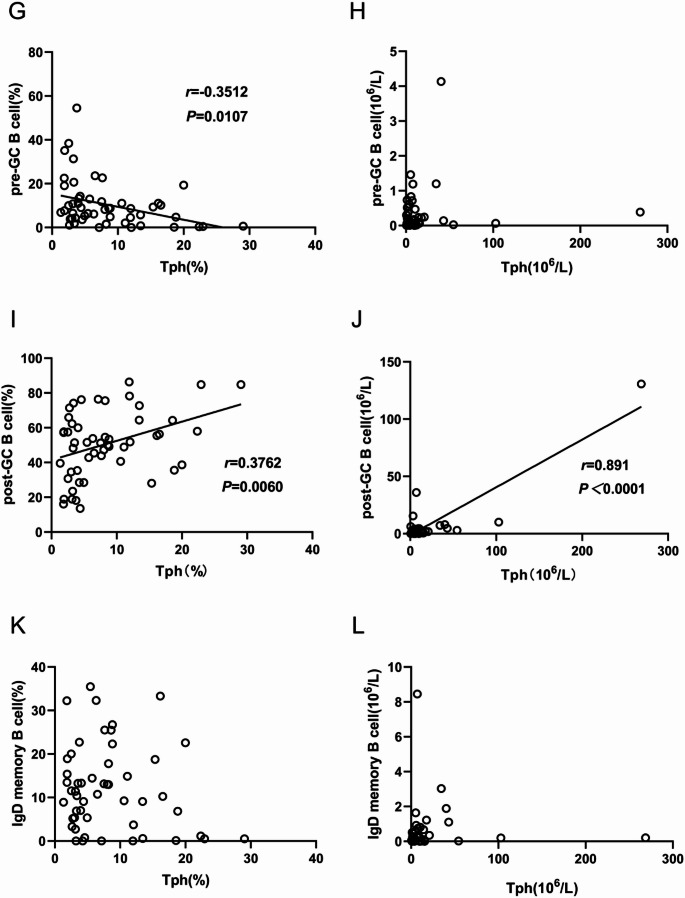
Correlation between B cells subsets and Tph cells expression. (**A-B**) Correlation between Tph cells expression and Naive B cells. (**C-D**) Correlation between Tph cells expression and Breg cells. (**E-F**) Correlation between Tph cells expression and plasma cells. (**G-H**) Correlation between Tph cells expression and pre-GC B cells. (**I-J**) Correlation between Tph cells expression and post-GC B cells. (**K-L**) Correlation between Tph cells expression and IgD memory B cells

We then explored the correlation between the B cells subsets and Tph cells in cGVHD patients. The percentage of Naive B cells, Breg cells and pre-GC B cells was negatively associated with Tph cells (Fig. 6A, C and G). The absolute number of plasma cells was positively associated with the absolute number of Tph cells (Fig. 6F). The percentage and absolute number of post-GC B cells were both positively associated with Tph cells expression (Fig. 6I and J). The results demonstrated that post-GC B cells expression were positively associated with Tph cells expression.

## Disscussion

In this study, we compared the presence and phenotype of Tph cells, as well as cTfh and B cells subsets among cGVHD patients, patients without cGVHD, and HCs. Tph cells, especially the activated and migratory Tph cells subsets, were increased in cGVHD patients. The dynamic changes of Tph cells, as well as the expression of activation and migration markers, during the onset and improvement of cGVHD suggested that they could be the biomarkers or therapeutic targets of cGVHD.

Allo-HSCT is currently an effective treatment for hematological system diseases. In recent years, the incidence of cGVHD remains high. Severe cGVHD affects the quality of life and even long-term survival of patients and is now an important cause of non-recurrent mortality in post-transplant patients [26–28]. The pathogenesis of cGVHD involves a complex process, and the clinical manifestations are diverse due to the widely distributed target organs and the great individual differences among patients, which makes the diagnosis and treatment of cGVHD extremely difficult. Therefore, it is particularly important to find a reasonable and effective biomarker to study the occurrence and development of cGVHD from the perspective of immune cells.

CD4^+^ T cells are central mediators in specific autoimmune diseases. Tfh cells, as a subset of CD4^+^ T cells, were first found in human tonsils and contribute to GC responses [29]. Tfh-mediated activation of B cells in the GC promotes differentiation of naive B cells into memory B cells and production of high affinity IgG, resulting in somatic hypermutation [30–32]. Our previous study showed that the percentage of Tfh cells in spleen was increased in the cGVHD mouse model [33]. Flynn et al. has found that the percentage of Tfh cells and GC B cells were significantly increased in murine cGVHD and concluded that Tfh cells were necessary for the formation of GC B cells and the production of Ig deposits [34]. Our results showed that the percentage of cTfh cells was significantly decreased in patients with cGVHD, which is consistent with the findings of Forcade et al. and Knorr et al. [35, 36]. Regarding the low percentage of cTfh cells in the cGVHD patients, Forcade et al. suggested that this may be due to abnormal homing and transport of cTfh cells in cGVHD patients, whereas Knorr et al. suggested that the cGVHD process itself causes the loss of cTfh cells.

Unlike Tfh cells, which interact with B cells within lymphoid organs, Tph cells provide help to B cells within inflamed tissues. Tph cells were originally identified in synovial tissue from patients with RA, but as circulating Tph cells have been increasingly studied, they have also been found to be associated with other autoimmune diseases such as SLE, T1D, psoriasis, and ulcerative colitis [13, 15, 21, 37]. The clinical presentation of cGVHD is like that of systemic autoimmune diseases. Rao et al. found that the proportion of Tph cells was independent of clinical parameters such as age, sex, duration of disease, but was only related to disease activity in RA [10]. Fortea-Gordo et al. had observed a significant reduction of Tph cells in early RA (eRA) patients who entered remission or achieved a significant clinical improvement, whereas cTfh cells level did not vary significantly in these patients [38]. Remarkably, we found a markedly increased percentage of Tph cells in the circulation of patients with cGVHD, compared with HCs. The percentage of Tph cells showed an increasing trend in patients with severe cGVHD compared to those with mild/moderate cGVHD. The absolute number of Tph cells was significantly higher in patients with severe cGVHD than in mild/moderate cGVHD, indicating that different cGVHD severities might attribute to different levels of Tph cells expression. We also found that the percentage and absolute number of Tph cells decreased significantly after anti-cGVHD treatment improved patients’ symptoms. This also demonstrated that Tph cells level were closely related to the progression of clinical cGVHD. In addition, ROC curve analysis also indicated that Tph cells was a risk factor for the development of cGVHD.

Previous study suggested that Tph cells co-expressing other activation markers, such as CD38 and MHC class II, were increased in patients with SLE [39, 40]. Besides, they also found a positive correlation between the frequency of CD38^+^Tph cells with the SLEDAI and anti-dsDNA antibody levels. The most remarkable difference between Tph cells and Tfh cells was a substantially distinct migratory program. Instead of expressing CXCR5 that enables Tfh cells to home to lymphoid follicles, Tph cells express a set of chemokine receptors associated with migration to inflamed peripheral tissues. Then we further measured the expression of Tph cells markers CD38 and CCR2 by flow cytometry in 22 post-transplant patients to assess the activation and migration status of Tph cells. Our study indicated that the percentage and absolute number of CCR2^+^Tph cells were higher in patients with severe cGVHD than in those with mild/moderate cGVHD and those without cGVHD. These findings suggest that the migratory status of Tph cells might reflects disease severity in cGVHD patients.

Imbalances of Tph cells subsets have been observed in SLE [39, 40]. Lin et al. reported that Tph1 cells are more abundant in SLE patients than in HCs [39]. Makiyama et al. found that Tph1 cells and Tph17 cells were both more abundant in SLE patients than in HCs and that enhanced activation status of Tph1 cells were associated with activated plasmablasts in SLE [40]. Phenotype of Tph17 cells is activated and proliferative in psoriasis vulgaris [21]. Tph17 cells and plasma CXCL13 could be biomarkers for evaluating the severity of psoriasis vulgaris. Our results suggested that the Tph cells subsets were dominated by an increased percentage and absolute number of Tph2 cells and Tph17 cells in cGVHD patients. Changes in Tph cells subsets indicated that there were imbalances of Tph cells subsets in cGVHD patients. Tph2 cells and Tph17 cells may be involved in the pathogenesis of cGVHD and suitable as biomarkers for assessing the severity of cGVHD.

To date, we have known that the percentage of Tph cells were elevated in autoantibody-positive RA as well as autoantibody-positive T1D patients [10, 15]. In addition, there are no remarkable change of Tph cells percentage in the peripheral blood of seronegative RA or spondyloarthropathy, which are negative for autoantibodies. The high level of Ig deposition in tissues of mouse cGVHD models and cGVHD patients, as well as the use of anti-CD20 monoclonal antibodies in refractory cGVHD, are sufficient to suggesting that B cells play an important role in tissue damage in cGVHD [5, 41]. Rao et al. demonstrated that Tph cells promoted plasma cell differentiation from memory B cells in a co-culture system [10]. Hou et al. revealed that Tph cells and B cells were expanded in muscle tissue in dermatomyositis patients, and the increased B cells accumulated around Tph cells [42]. Together, all these suggesting that the mechanism of Tph cells involvement in the disease may be related to B cells. Previous mouse models and clinical studies have shown that the development of cGVHD is associated with a variety of B cells reconstitution abnormalities. Our results found decreased percentage of Naive B cells, but increased percentage of pre-GC B cells in post-transplant patients compared to HCs. This is consistent with the Sarantopoulos’ findings that the onset of cGVHD may be closely linked to a delay in the Naive B cells remodeling [22]. Additionally, we found that the presence of Tph cells was positively correlated with post-GC B cells. However, only the percentage of Tph cells was negatively correlated with Naive B cells, Breg cells, and pre-GC B cells. These suggested that Tph cells do not need to upregulate the expression of CXCR5, that is, they do not need to combine CXCR5 with CXCL13 to directly assist B cells activation, thus aggravating cGVHD, which still needs to be further verified by co-culture of Tph cells and B cells in vitro.

In conclusion, we found increased Tph cells and CCR2^+^Tph cells in cGVHD patients than in patients without cGVHD, which correlated with disease severity. The percentage and absolute number of Tph cells decreased significantly after the improvement of patients’ symptoms with anti-cGVHD treatment compared to the pre-treatment period. Tph cells subsets in cGVHD patients are dominated by an increased proportion of Tph2 cells and Tph17 cells population. Overall, Tph cells are involved in the onset and progression of cGVHD and correlates with the severity of the disease. Our study shed light on the clinical significance of Tph cells as a potential candidate for developing a biomarker of cGVHD progression and a therapeutic target of cGVHD treatment in the future.

## Supplementary Information

Below is the link to the electronic supplementary material.

Supplementary Material 1

## Data Availability

No datasets were generated or analysed during the current study.
