# Supplementary material for: Expansion of peripheral helper T cells in the peripheral blood of patients with chronic graft-versus-host disease
Source: Ann Hematol. 2025 Nov 3;104(10):5343–58. doi: 10.1007/s00277-025-06663-w (PMC12619759; doi:10.1007/s00277-025-06663-w)
Supplement: Supplementary file 1 — Supplementary Material 1 [file 277_2025_6663_MOESM1_ESM.docx]

Table S1 B-cell subset phenotypes

| CD19^+^B cell subset | Phenotype |
| --- | --- |
| CD27^-^B cell subset |  |
| Naive B | CD19^+^CD27^-^CD38^-^IgD^+^ |
| Breg | CD19^+^CD27^-^CD38^+^IgD^+^ |
| CD27^+^B cell subset |  |
| IgD memory B | CD19^+^CD27^+^CD38^-^IgD^+^ |
| Pre-GC B | CD19^+^CD27^+^CD38^+^IgD^+^ |
| Post-GC B | CD19^+^CD27^+^CD38^-^IgD^-^ |
| Plasma | CD19^+^CD27^+^CD38^+^IgD^-^ |
